# Deoxynivalenol Exposure Assessment for Pregnant Women in Bangladesh

**DOI:** 10.3390/toxins7103845

**Published:** 2015-09-24

**Authors:** Nurshad Ali, Meinolf Blaszkewicz, Abdullah Al Nahid, Mustafizur Rahman, Gisela H. Degen

**Affiliations:** 1 Leibniz Research Centre for Working Environment and Human Factors (IfADo) at TU Dortmund, Ardeystr. 67, Dortmund D-44139, Germany; E-Mails: blaszkewicz@ifado.de (M.B.); degen@ifado.de (G.H.D.); 2 Department of Biochemistry and Molecular Biology, Rajshahi University, Rajshahi 6205, Bangladesh; 3 Department of Biochemistry, Gonoshasthaya Samaj Vittik Medical College and Hospital, Gono University, Dhaka 1344, Bangladesh; E-Mail: m22_rahman@yahoo.com; 4 Department of Physiology, Gonoshasthaya Samaj Vittik Medical College and Hospital, Gono University, Dhaka 1344, Bangladesh; E-Mail: drsalnahid79@gmail.com

**Keywords:** deoxynivalenol, deepoxy-deoxynivalenol, exposure, mycotoxins, urine

## Abstract

The trichothecene mycotoxin deoxynivalenol (DON) is a contaminant of crops worldwide and known to cause adverse health effects in exposed animals and humans. A small survey reported the presence of DON in maize samples in Bangladesh, but these data are insufficient to assess human exposure, and also, biomonitoring data are still scarce. The present study applied biomarker analysis to investigate the DON exposure of pregnant women in Bangladesh. Urine samples were collected from pregnant women living in a rural (*n* = 32) and in a suburban (*n* = 22) area of the country. Urines were subjected to enzymatic hydrolysis of glucuronic acid conjugates and to immunoaffinity column clean-up prior to LC-MS/MS analysis of DON and its de-epoxy metabolite DOM-1. The limits of detection (LOD) for DON and DOM-1 in urine were 0.16 ng/mL and 0.10 ng/mL, respectively. DOM-1 was not detected in any of the urines, whilst DON was detectable in 52% of the samples at levels ranging from 0.18–7.16 ng/mL and a mean DON concentration of 0.86 ± 1.57 ng/mL or 2.14 ± 4.74 ng/mg creatinine. A significant difference in mean urinary DON levels was found between the rural (0.47 ± 0.73 ng/mL) and suburban (1.44 ± 2.20 ng/mL) cohort, which may be related to different food habits in the two cohorts. Analysis of food consumption data for the participants did not show significant correlations between their intake of typical staple foods and DON levels in urine. The biomarker concentrations found and published urinary excretion rates for DON were used to estimate daily mycotoxin intake in the cohort: the mean DON intake was 0.05 µg/kg b.w., and the maximum intake was 0.46 µg/kg b.w., values lower than the tolerable daily intake of 1 µg/kg b.w. These first results indicate a low dietary exposure of pregnant women in Bangladesh to DON. Nonetheless, further biomonitoring studies in children and in adult cohorts from other parts of the country are of interest to gain more insight into DON exposure in the population of Bangladesh.

## 1. Introduction

Deoxynivalenol (DON) is a trichothecene mycotoxin produced by *Fusarium* species, mainly *F. graminearum* and *F. culmorum*, and the most frequently-detected mycotoxin contaminant of maize, wheat and barley in temperate regions of the world [1,2]. Consumption of DON-contaminated feed has been associated with a number of adverse effects in animals, including feed refusal, vomiting, diarrhea, dizziness and fever, and chronic exposure to DON can lead to growth faltering, immunological and neurological dysfunction [3,4]. The acute effects of DON (or “vomitoxin”) in humans are similar to those seen in animals, and chronic dietary exposure of animals to DON causes altered nutritional efficiency [2,5], whilst long-term effects in humans have not been established so far. The primary toxic effect of DON is inhibition of protein synthesis, and it activates a signaling pathway known as ribotoxic stress response in cells and induces apoptosis [4,6]. Moreover, the effects include altered neuroendocrine signaling, impaired gut integrity and immune function [7,8].

DON is thus a concern for human health, and exposure early in life is of special interest in light of the following: DON transfer via the placenta to the fetus occurs in sows [9], and maternal exposure has been linked to growth retardation [10] and immunosuppression in the offspring [11]. Since DON has been shown to cross also the human placenta, dietary exposure during pregnancy will lead to DON exposure of the fetus, whose detoxification mechanisms are not yet well developed [12].

Along with conventional mycotoxin analysis in food, biomonitoring has greatly advanced an assessment of human DON exposure. As reviewed by Turner *et al.* (2012) [13], sensitive analytical methods for the analysis of DON and its metabolites in urine have been developed and validated as biomarkers of exposure: unmetabolized or “free” DON together with DON-glucuronides in urine reflect rather strong dietary mycotoxin exposure, and the mean estimated amount of biomarkers excreted within a day are rather high, with about 68% [14] and 72% of the DON intake [15]. As DON is largely present as a glucuronide conjugate in human urine [14,15], enzymatic hydrolysis of samples (deconjugation) is used in most single- or multi-analyte methods for biomarker determination to increase the detectability of total DON [16]. Analysis should also include DOM-1, the detoxification product of DON formed by gut microbiota in animals and humans: a recent study found relatively high levels of DOM-1, indicating that a substantial proportion of DON can be detoxified in humans [17], whilst the DOM-1 metabolite has been rarely detected or at only very low levels in urines from other cohorts [15,18,19,20].

DON biomarker occurrence has been analyzed in the urines of pregnant women from the U.K., Egypt and Croatia [21,22,23]. More studies have been conducted in the general population of some Asian [19,24], European [15,18,20,25,26,27,28,29,30] and African countries [31,32]. Overall, the results from these biomonitoring studies (in terms of frequency of detection and urinary analyte levels) indicate quite variable human exposure to DON, a finding in accord with reported differences in DON contamination of food commodities in various regions of the world [1,6].

When food contaminant data are scarce, as often is the case in developing countries, analysis of biomarkers in human body fluids provides useful insights, since biomonitoring covers mycotoxin intake from all dietary sources and exposure by various routes [14,33]. In Bangladesh, a small survey detected the presence of DON in 10 maize samples (17% positive) with the highest level of 337 µg/kg in maize from the northern part of the country [34]. The reported DON levels did not exceed the U.S. or EU regulatory limits, but this survey focused on maize alone and did not include analysis of other possibly contaminated food commodities. In short, these data are insufficient to assess human DON exposure in Bangladesh.

Therefore, to gain more insight into maternal DON exposure during pregnancy, urine samples were collected from inhabitants of a rural and a suburban area of the Savar region in the Dhaka district of Bangladesh. The present study is the first biomarker-based DON exposure assessment for pregnant women in Bangladesh.

## 2. Results

### 2.1. Validation Parameters for Biomarker Analysis

The calibration curve with pure standards in the mobile phase showed linearity in the range of 0.5–20 ng/mL for both analytes with a coefficient of determination (*R*^2^) of 0.998 for DON and 0.997 for DOM-1, respectively. Analysis of DON and DOM-1 spiked urine yielded similar data for linearity in the range of LOD, 10 ng/mL. The limit of detection (LOD) and limit of quantification (LOQ) were determined based on the lowest quantity of analyte that can be clearly distinguished from the background (LOD; signal to noise ratio, S/N = 3) or quantified (LOQ; S/N ≥ 6). For DON, the LOD was 0.16, and the LOQ was 0.30 ng/mL. For DOM-1, the LOD and LOQ were 0.10 and 0.20 ng/mL, respectively.

Recovery assays were performed in a urine with no measurable background of DON and DOM-1. Isotope-labeled internal standard ([^13^C_15_] DON) was used to correct for recovery. Recovery of analytes was assessed at three concentration levels in triplicates of spiked samples with mean values of 93% and 86% for DON and DOM-1, respectively (Table 1). The intra-day and inter-day repeatability (Table 2) at a spike concentration of 1.0 ng/mL (*n* = 6) were determined using also the mycotoxin-free urine and showed acceptable precision for the measurements.

**Table 1:** Recovery of DON and DOM-1 in human urine.

Spike Level (ng/mL)	DON		DOM-1	
	Recovery (%)	RSD (%)	Recovery (%)	RSD (%)
0.5 (*n* = 3)	102	12.2	88	8.5
1.0 (*n* = 3)	90	8.4	92	4.8
2.0 (*n* = 3)	94	10.4	78	4.2

**Table 2:** Intra-day and inter-day assay repeatability for DON and DOM-1 in urine.

Spike Level (ng/mL)	DON			DOM-1		
	Mean ± SD (ng/mL)	Recovery (%)	RSD (%)	Mean ± SD (ng/mL)	Recovery (%)	RSD (%)
Intra-day (1.0) *n* = 6	0.92 ± 0.12	92	13.0	0.82 ± 0.06	82	7.3
Inter-day (1.0) *n* = 6	0.88 ± 0.08	88	9.1	0.80 ± 0.08	80	10.0

### 2.2. Demographic Characteristics of the Participants

The baseline anthropometric data of the participants are summarized in Table 3. The mean age of the participants was 25 ± 5 years, ranging from 18–36 years with no significant difference between the rural and suburban cohort. The average body mass index (BMI) for all subjects was 20.1 ± 3.0 kg/m^2^. Volunteers of the rural cohort had significantly higher BMI (21.2 ± 2.3 kg/m^2^) than those in the suburban cohort (18.6 ± 3.2 kg/m^2^). There were no significant differences (*p* < 0.01) in urinary creatinine levels between the rural (652 ± 509 mg/L) and suburban (710 ± 472 mg/L) cohort. Regarding occupation, 83% of the participants were housewives, and 17% were office workers. All participants consumed the typical Bangladeshi staple food rice and, to a lesser extent, roti (whole wheat flatbread); these are generally consumed with vegetables, lentils, fish, poultry and beef.

**Table 3:** Demographic characteristics of the participant.

Characteristics	Rural	Suburban	Total
*n*	31	22	54
Age (years)			
Mean ± SD	25 ± 5	26 ± 5	25 ± 5
Range	18–36	18–36	18–36
Occupation (*n*, %)			
Housewives	29 (91)	16 (73)	45 (83)
Office workers	3 (9)	6 (27)	9 (17)
BMI (kg/m^2^)			
Mean ± SD	21.2 ± 2.3 *	18.6 ± 3.2	20.1 ± 3.0
Range	14.3–28.2	12.0–24.4	12.0–28.2
Creatinine (mg/L)			
Mean ± SD	652 ± 509	710 ± 472	676 ± 490

* *p* < 0.01 when compared to suburban; *p*-value obtained from independent sample *t*-test.

### 2.3. Urinary Level of DON in the Cohorts

**Figure 1:**
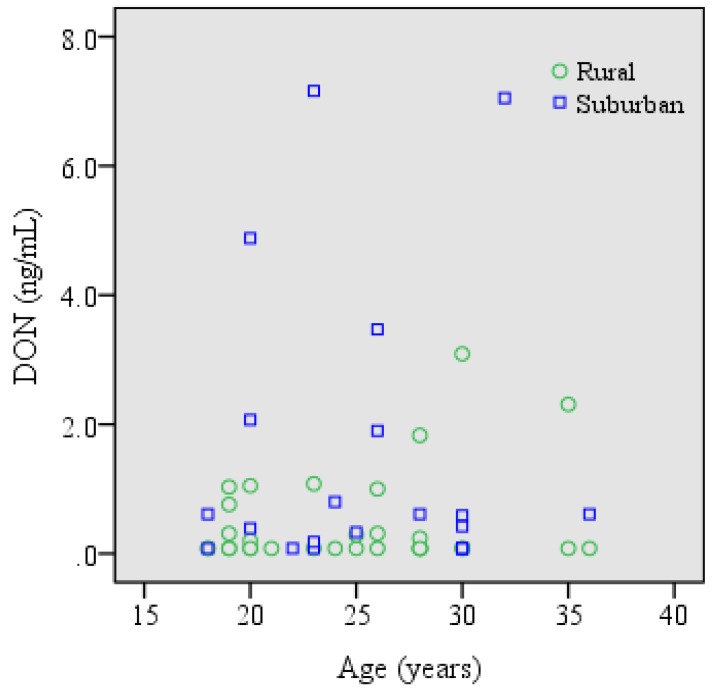
Distribution of urinary DON in the rural and suburban cohort. Biomarker concentration was set to half of the LOD for samples with DON below the LOD.

Urines with analyte concentrations at or above the limit of detection (LOD) were considered as positive samples. DOM-1 was not detected in any of the urines, whilst DON was detectable in 52% of the urines, at concentrations ranging from 0.18–7.16 ng/mL (Table 4). The distribution of urinary DON levels according to age of both cohorts is presented in Figure 1. The mean concentration of urinary DON was 0.86 ± 1.57 ng/mL or 2.14 ± 4.74 ng/mg creatinine for all study participants. A significant difference (*p* < 0.05) in mean urinary DON levels was found between the rural (0.47 ± 0.73 ng/mL) and the suburban (1.44 ± 2.20 ng/mL) cohort (Table 4), which may be related to different food habits in the two cohorts. Statistical analysis revealed no significant correlation of urinary biomarkers with age, gender and BMI of the study subjects.

**Table 4:** Occurrence and contamination levels of DON in urine.

Cohort	*n*	Positive Samples *n* (%)	Mean ± SD (ng/mL)	Median (Range) (ng/mL)	75th Percentile (ng/mL)	Mean ± SD (ng/mg Creatinine)
Rural	32	13 (41)	0.47 ± 0.73	nd (nd–3.09)	0.65	1.14 ± 2.47
Suburban	22	15 (68)	1.44 ± 2.20 *	0.51 (nd–7.16)	1.94	3.60 ± 6.63
Total	54	28 (52)	0.86 ± 1.57	0.19 (nd–7.16)	0.85	2.14 ± 4.74

A positive sample refers to urines containing the analyte ≥LOD; nd refers to levels below the LOD. For the calculation of mean and median values, biomarker concentration was set to half of the LOD if the DON urine concentration was below the LOD. * p < 0.05 when compared to rural; *p*-value obtained from independent sample *t*-test.

### 2.4. Estimation of DON Intake

Using the urinary biomarker analysis data, the provisional daily DON intake among all participants was on average 52.5 ± 94.4 ng/kg body weight (b.w.), and the highest DON intake was 461 ng/kg b.w. (Table 5). A significant difference (*p* < 0.05) was found between the rural (27 ng/kg b.w.) and suburban (90 ng/kg b.w.) cohort. No individual had an estimated daily DON intake above the tolerable daily intake (TDI) of 1000 ng/kg b.w./day [1].

**Table 5:** Provisional daily intake (PDI) of DON ^a^. TDI, tolerable daily intake.

Cohort	*n*	Mean ± SD ng/kg b.w.	Median ng/kg b.w.	Range ng/kg b.w.	Exceeding TDI *n* (%)
Rural	32	26.8 ± 39.0	5.0	3.1–154.0	-
Suburban	22	89.9 ± 133.2 *	31.1	4.7–460.8	-
Total	54	52.5 ± 94.4	11.2	3.1–460.8	-

^a^ Dietary DON intake was calculated based on urinary DON concentrations, adjusted for 24-h urine volume, assuming a 68% clearance rate and individual body weight (see the Experimental Section for details). * *p* < 0.05 when compared to rural; *p*-value obtained from independent sample *t*-test.

### 2.5. Correlation between Urinary DON and Food Intake

Based on information provided in the food frequency questionnaires (FFQ), possible correlations were analyzed between food consumption (graded 1–4) and urinary DON levels in the pregnant women cohort. The food items rice, wheat/maize, lentils and chicken meat were considered in Spearman correlation analysis (Table 6). Consumption of rice, the main staple food in Bangladesh, did not show a significant correlation (*p* = 0.39) with the urinary DON level of the subjects. No significant correlations were observed between DON in urine and other food items, such as wheat/maize (*p* = 0.75), lentil soup (*p* = 0.37) or chicken meat (*p* = 0.46).

**Table 6:** Correlation (*r*) between food consumption and DON concentration in urine.

Foodstuff	Correlation (*r*)	*p*-Value
Rice	−0.174	0.395
Wheat/maize	0.067	0.746
Lentil soup	0.182	0.374
Chicken meat	0.151	0.462

Only positive samples (≥LOD) are considered for the calculation. Assessment of food consumption was done using the score 1–4 (1 = do not consume at all, 2 = 1 time daily, 3 = 2 times daily, 4 = 3 times daily. *p-*values are obtained from Spearman’s correlation coefficient (two-tailed).

## 3. Discussion

As briefly outlined in the Introduction, DON is known to cause a range of adverse effects in animals and can also cross the placenta. Thus, exposure of pregnant women to DON is of particular concern, yet only few biomonitoring studies have been conducted so far in this group of the population.

The present study is the first biomarker survey for DON exposure of pregnant women in Bangladesh: frequent detection of DON (52%) at a mean concentration of 0.86 ± 1.57 ng/mL or 2.14 ± 4.74 ng/mg creatinine (Table 4) indicates DON exposure in pregnant women living in a rural and a suburban area of Bangladesh, albeit at comparatively moderate levels. The urinary DON concentrations determined in this study are similar to levels found in the urines of pregnant women from Egypt (geometric mean 1.11 ng/mg creatinine, range 0.5–59.9 ng/mg creatinine [22]). On the other hand, values in our cohort (mean 2.1 ng/mg creatinine, range 0.6–24.3 ng/mg creatinine) are clearly lower than those reported for pregnant women (*n* = 85) from Bradford, the U.K. (geometric mean 10.3 ng/mg creatinine, range 0.5–116.7 ng/mg creatinine [21]) and for pregnant women (*n* = 40) from Croatia (DON equivalents: mean 111.8 ng/mL, range 4.8–1238 ng/mg [23]). The authors noted that maximum concentration in the rural sub-cohort of Croatian pregnant women indicated a nine-times higher exposure to DON than in U.K. pregnant women. Moreover, biomarker-based calculation of DON intake revealed mycotoxin exposures that clearly exceeded the tolerable daily intake (TDI) for DON of 1 µg/kg b.w., both in the U.K. and the Croatian cohort [21,23]. In the Bangladeshi cohort of pregnant women, even the highest calculated provisional daily DON intake of 461 ng/kg b.w. (Table 5) did not exceed the TDI.

In our survey, DOM-1 metabolite was not detected in any of the 54 Bangladeshi urines. This is of interest, since human data on the occurrence of this detoxication product are limited. DOM-1 was found at modest levels in urines from Egyptian pregnant women (2/69; [22]) and from French farmers (26/76; [18]), but it was not detected in studies of U.K. adults [20] or U.K. pregnant women [21]. However, in a recent small pilot study with 15 volunteers who provided spot urines in two years, DOM-1 was detected in 37% and 40% of the samples: The prevalence and the proportion of DOM-1 (up to 17.8% of urinary DON in some individuals) indicated that a substantial fraction of DON can be detoxified in humans harboring gut microbiota capable of DON detoxication [17]. Other recent studies from Europe detected DOM-1 less frequently and also at very low levels compared to DON and DON glucuronide [28,35,36]. DON toxicity in humans may be also related to their ability to produce DON-glucuronide [37]. Yet, in light of the limited availability of urines, we did not determine the ratio of free DON (aglycone) to DON-glucuronides in our cohort.

Regarding possible sources of DON intake in Bangladesh: analysis of correlations between urinary biomarker levels and food consumption pattern based on FFQ data did not show significant associations with typical food items (Table 6), and this may be due to the overall rather low DON exposure among the participants. Interestingly, pregnant women from the suburban area had a significantly higher mean level of urinary DON and also provisional daily DON intake than our rural cohort (Table 4 and Table 5). The higher DON exposure in the suburban participants may be related to higher consumption of wheat bread by the suburban residents than the rural residents. However, maize cannot be excluded, since our FFQ did not assess wheat and maize consumption separately, and wheat and maize flours are sometimes mixed together for making homemade bread and also in the bakery industry in Bangladesh. A small survey reported DON contamination at a low level in maize samples collected from the northern part of Bangladesh [34], but food analysis data for DON are not available for other staple food, such as rice or wheat.

Finally, the present biomonitoring study revealed only moderate exposure of pregnant women in Bangladesh. Nonetheless, further studies are indicated: urine samples were collected in wintertime (2014), but the country has a subtropical monsoon climate, characterized by clear seasonal variations in rainfall, temperature and humidity (http://en.wikipedia.org/wiki/Geography_of_Bangladesh). Since the growth and distribution of *Fusarium* fungi depends on climatic factors, such as moisture and temperature, quite variable DON contamination of crops cannot be excluded [17,38].

## 4. Experimental Section

### 4.1. Standards and Reagents

Standards for deoxynivalenol (DON), de-epoxy DON (DOM-1) and isotope-labeled internal standard ([^13^C_15_] DON) were obtained from Romer Labs Diagnostics GmbH (Tulln, Austria). The enzyme β-glucuronidase/arylsulfatase (β-Gluc/ArylS) from *Helix pomatia* (with specific activity 5.5 U/mL β-glucuronidase, 2.6 U/mL arylsulfatase at 37 °C) was from Roche Diagnostics (Mannheim, Germany) and used with 10-fold hydrolysis buffer (13.6 g sodium acetate hydrate, 1.0 g ascorbic acid, 0.1 g EDTA in 100 mL deionized water, adjusted to pH 5.0 with acetic acid 98%) for enzymatic treatment of urine samples. Immunoaffinity DONTest™ columns (Vicam^®^, purchased from Ruttmann, Hamburg, Germany) were used for clean-up and enrichment of analytes. Methanol (LC-MS grade) was purchased from Merck (Darmstadt, Germany).

### 4.2. Participants and Sample Collection

During February–March 2014, fifty-four pregnant women (*n* = 47 in the third, *n* = 6 in the second and *n* = 1 in the first trimester) were recruited from a rural (Dhamrai, Dhamsona, Nolam, Paichail) and a suburban area (Baipail, Dhamrai, Kashimpur, Modhupur, Namabazar) of the Savar region in the Dhaka district of Bangladesh. All participants were apparently healthy according to external clinical examination by professional nurses. Written consent was obtained from the women before inclusion in this study. Volunteers were asked to fill out a short questionnaire for anthropometric information (age, height and weight), occupation and food habits, using the same food frequency questionnaire (FFQ) as in our previous studies in Bangladesh [39]. The spot urine samples were collected in the morning (between 8:00 and 11:00 am) into non-sterile disposable containers and then stored at −20 °C. All urine samples were shipped on dry ice to IfADo (Dortmund, Germany) in March 2014, and biomarker analysis was conducted in January 2015. This study was approved by the Institute of Biological Sciences of Rajshahi University, Rajshahi-6205, Bangladesh (Memo no. 40/320/IAMEBBC/IDSC), and by the Institutional Internal Review Board of IfADo.

### 4.3. Enzymatic Hydrolysis

To cleave DON (and DOM-1) conjugates, 250 µL hydrolysis buffer (pH 5.0) and 40 µL of β-Gluc/ArylS enzyme were added to 3 mL urine aliquots and incubated at 37 °C overnight before sample extraction.

### 4.4. Sample Preparation

Urine sample clean-up and enrichment of analytes was performed by immunoaffinity column (IAC) extraction with DONTest™ following the protocol provided by the manufacturer (VICAM). Briefly, after rinsing the column with 1 mL of water, the entire hydrolyzed urine sample was loaded on a DONTest™ column at a flow rate of 1 drop/s. The column was washed with 5 mL distilled water, then DON was eluted (flow rate 1 drop/s) from the column using 2 mL of methanol. The elute was evaporated to dryness under a stream of nitrogen at 45 °C; the residue was dissolved in 500 µL water/methanol (90:10), vortexed and filtered through a 0.45-µm pore size Teflon syringe filter prior to LC-MS/MS analysis. Thus, the enrichment factor was 6.

### 4.5. LC-MS/MS Analysis

DON and its metabolite DOM-1 were measured in urine extracts by liquid chromatography with tandem mass spectrometry. Analysis was performed with a Varian 1200-L Quadrupole MS/MS equipped with an electrospray ionization (ESI) source, a Prostar^®^ Varian HPLC system and a Varian MS Workstation version 6.9.1 data system (Agilent Technologies, Germany). The following settings were used: nitrogen as the drying gas (21 psi), gas temperature 250 °C, and argon used as the collision gas (2.0 mTorr). Chromatographic separation was carried out at 25 °C on a Nucleosil^®^ 100-5 C_18_ 125 × 3 mm column (Macherey-Nagel, Dŭren, Germany) with water (Mobile Phase A) and methanol (Mobile Phase B) as eluents in the following gradient: 0–1.1 min 45% B, 1.1–5.3 min 60% B, 5.3–7.3 min 95% B (column wash), 8–18 min 45% B (re-equilibration). The flow rate was 0.2 mL/min, and the injection volume was 20 µL. The retention time for DON and its metabolite DOM-1 was 4.5 and 5.6 min and for the internal standard (IS) 4.5 min. ESI-MS/MS was executed by multiple reaction monitoring (MRM) in negative ion mode. The specific transitions of precursor ion and product ion were as follows: 295.1 → 265.1 *m*/*z* and 295.1 → 138.1 for DON. The optimized collision energy (CE) was −10 and −15.5 eV, respectively. For DOM-1, the transitions of precursor and product ions were 279.1 → 248.9 and 279.1 → 231.1 *m*/*z* with an optimized CE of −9 and −13 eV, and for the internal standard (IS), the transition of precursor and product ions was 310.0 → 279.2 *m*/*z* with an optimized CE of −9 in that order.

### 4.6. Creatinine Analysis

Urinary creatinine was measured by a modified Jaffe method on a 96-well plate reader (Tecan Genios) [40] to account for variability in urine dilution between individual samples. Urinary DON levels determined in ng/mL were adjusted for creatinine in the urine sample and their concentrations expressed as ng/mg creatinine to allow also comparison with other biomarker data.

### 4.7. Exposure Assessment

The estimation of DON intake among the participants was performed based on the results of urinary DON analysis. The following equation was used to assess the provisional daily intake (PDI) of DON among the participants:

(1) $$PDI\left(\frac{\text{μg}}{\text{Kg}}\text{bodyweight}\right)=\frac{C\;\times V\;\times 100}{W\;\times E}$$

with *C* = biomarker concentration (µg/L), *V* = daily urine excretion (L), *W* = body weight (kg) and *E* = excretion rate (%). In the calculation, urinary output during pregnancy was considered as 2 L per day [41]. Individual body weight was used during the calculation of PDI and a daily urinary DON excretion rate of 68% [14], a value close to estimates in other studies [15,42].

### 4.8. Food Consumption Data

All participants were asked to fill in a questionnaire with a focus on the last two days of food consumption prior to urine sampling and in addition to record their regular food habits. The food frequency questionnaire (FFQ) asked for the intake of typical food items consumed by Bangladeshi people: staple foods, mainly cereals, such as rice, wheat, maize and lentils, as the major pulses. Consumption frequency was graded 1–4 (see Table 4). Chicken meat, eggs, groundnuts, milk and milk-based products were also included. Among these items, only rice is regularly consumed one to three times in a day and, to a lesser extent, also roti (whole wheat flatbread) for breakfast by the majority of participants.

### 4.9. Statistical Analysis

Descriptive statistics are presented as means (±SD), medians and interquartile ranges. Those samples containing DON below the limit of detection (LOD) were assigned a value of one-half the detection limit for the calculation of mean and median values, since this is considered a better approach to estimate average concentrations for left censored data, rather than assigning a value of zero to measurements below the detection limit [43]. Differences in urinary DON levels between the rural and the suburban cohort were analyzed by an independent sample *t*-test. The Spearman correlation coefficient (two-tailed) was used to assess the correlation between urinary DON concentration with food consumption, age and body mass index (BMI) of the participants. All analyses were carried out using IBM SPSS Statistics Version 22. A level of alpha 0.05 was assigned for statistical significance.

## 5. Conclusions

DON exposure in pregnant women in Bangladesh appears to be modest and lower than observed in biomonitoring studies performed in Europe and Africa. However, the present investigation comprises analysis of urine samples collected in one district and season only. Therefore, further studies in children and adult cohorts from other parts of the country and another season are indicated to gain more insights into the DON exposure of the Bangladeshi population.
